# Soil microbiome dataset from Yok Don national park in the Central Highlands region of Vietnam

**DOI:** 10.1016/j.dib.2022.107798

**Published:** 2022-01-05

**Authors:** Dinh Minh Tran, To Uyen Huynh, Thi Huyen Nguyen, Tu Oanh Do, Thi Phuong Hanh Tran, Quang-Vinh Nguyen, Anh Dzung Nguyen

**Affiliations:** a Institute of Biotechnology and Environment, Tay Nguyen University, Buon Ma Thuot, Dak Lak 630000, Viet Nam; b Faculty of Natural Science and Technology, Tay Nguyen University, Buon Ma Thuot, Dak Lak 630000, Viet Nam

**Keywords:** Soil microbiome, Metagenomic next-generation sequencing, Yok Don national park, The dry deciduous dipterocarp forest

## Abstract

The Central Highlands region contains most of the national parks in Vietnam with different ecosystems, including the national parks of Kon Ka Kinh, Chu Mon Ray, Chu Yang Sin, Yok Don, Bidoup-Nui Ba, and Ta Dung. Thus, this region is considered a center with the highest biodiversity in Vietnam [1]. Among the national parks, Yok Don is unique in its conservation of the dry deciduous dipterocarp forest. Furthermore, Yok Don is the second-largest park in Vietnam; it has the most different ecosystem compared with other national parks in this region [2]. Although some studies have investigated biodiversity preservation in the region, some other studies have only dealt with medicinal plants, lichens, and the rhizospheric bacteria of cultivated black pepper [1,[3], [4], [5]. To the best of our knowledge, no research on the microbial communities in Yok Don national park and in the Central Highlands has been reported. At present, global warming and a decrease in the forest area in the Central Highlands have led to the ongoing reduction in biodiversity and microbial resources.

The current study reports the microbiome dataset from the soil sample collected in Yok Don national park. Metagenomic next-generation sequencing was used to characterize the microbial communities in the sample. The metagenome dataset generated provides information on microbial diversity and its functionality and can be useful for further studies on the conservation and use of microbial genetic resources in this region.

## Specifications Table

**Table:** 

Subject	Microbiology: *Microbiome*
Specific subject area	Metagenomics
Type of data	Figures, Tables, and Fastq files
How the data were acquired	Illumina MiSeq platform
Data format	Raw and Analyzed
Description of data collection	A soil sample was collected from Yok Don national park in the Central Highlands, Vietnam. Total DNA was extracted from the sample, and 16S rRNA gene amplicon sequencing was performed using the Illumina MiSeq platform (2 × 150-bp paired ends)
Data source location	• Institution: Yok Don national park • District/Province/Region: Buon Don, Dak Lak, the Central Highlands • Country: Vietnam • Latitude and longitude coordinates for collected samples: 12°58′22.82′′N,107°49′13.96′′E
Data accessibility	Data are available at the NCBI with Bioproject PRJNA783494 and SRA accession number SRR17036647 (https://trace.ncbi.nlm.nih.gov/Traces/sra/?run=SRR17036647)

## Value of the Data

• The data generated provides information on the microbiome in the soil at Yok Don national park in the Central Highlands, Vietnam.
• The data could be useful for the comparative analysis of the taxonomic profiles of Yok Don national park with those of other national parks.
• The data could be useful for future studies on the conservation and use of indigenous microbial gene resources for sustainable crop production and related fields.

## 1 Data Description

**Fig. 1:**
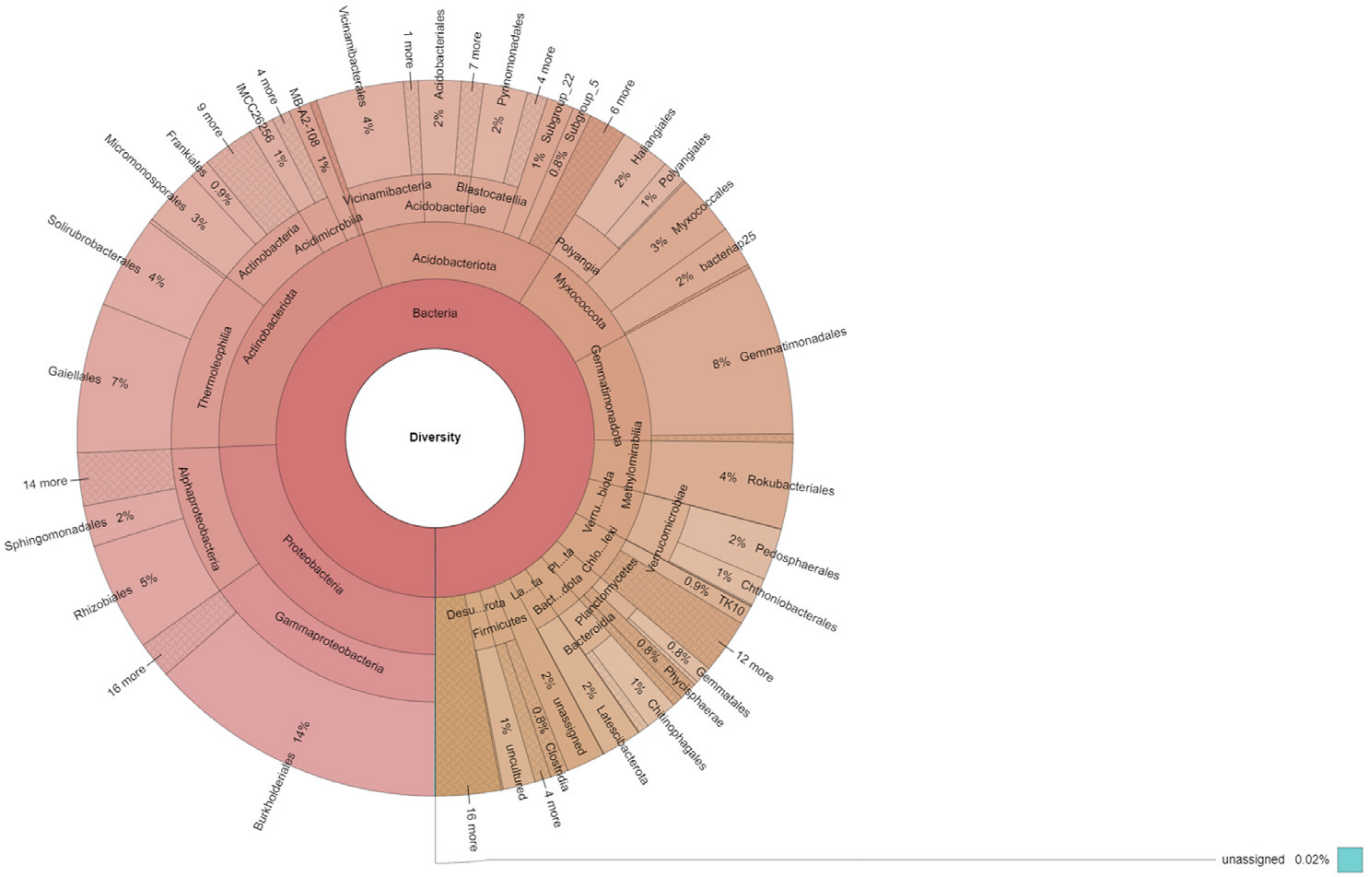
Taxonomic profile based on the 16S rRNA gene amplicon sequencing of the soil sample collected from Yok Don national park in the Central Highlands, Vietnam.

**Fig. 2:**
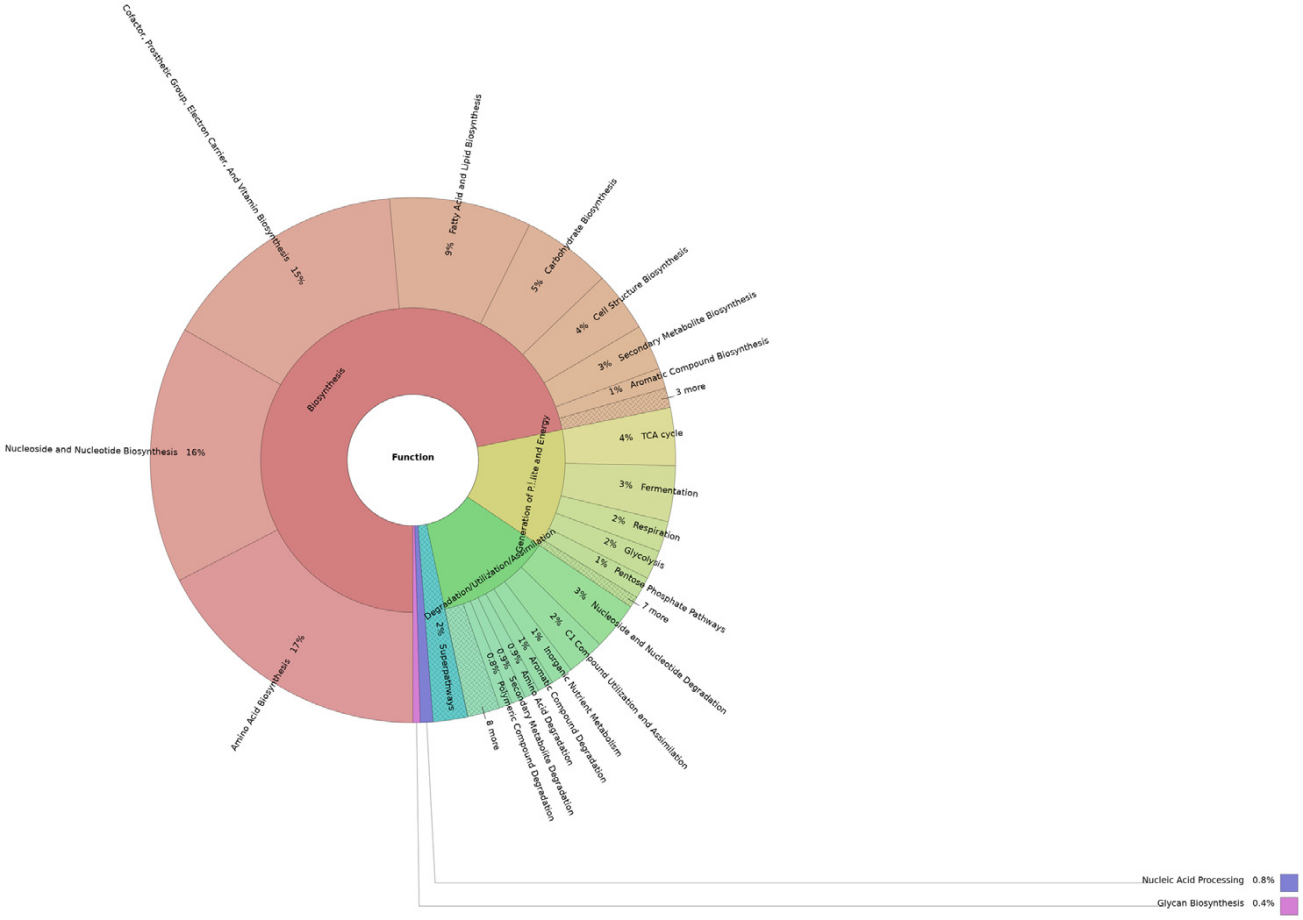
Functional profile based on the 16S rRNA gene amplicon sequencing of the soil sample collected from Yok Don national park in the Central Highlands, Vietnam.

The dataset describes the taxonomic and functional profiles of a metagenomic soil sample collected from Yok Don national park in the Central Highlands, Vietnam. The 16S rRNA gene amplicon sequencing was performed using the Illumina MiSeq platform (2 × 150-bp paired ends). Data were analyzed using classify-consensus-blast from QIIME2 aligned with the SILVA SSURef reference database (v.138), PICRUSt2, and MetaCyc database. A total of 190,918 reads were classified out of 190,953 analyzed reads (Table 1). Data were presented as taxonomic and functional profiles, as shown in Figs. 1 and 2, respectively. Among the 29 phyla detected, Proteobacteria (24.33%) was the most dominant, followed by Actinobacteriota (20.28%), Acidobacteriota (14.26%), Myxococcota (8.23%), and Gemmatimonadota (8.09%) (Fig. 1). Of the 188 bacterial orders present, Burkholderiales (13.53%) was the most abundant, followed by Gemmatimonadales (7.7%), Gaiellales (6.8%), Rhizobiales (4.92%), and Solirubrobacterales (4.19%). Moreover, 263 families and 380 genera were identified. Additionally, biosynthesis (71.78%) was the most abundant metagenomic function of the microbiome, followed by the generation of precursor metabolite and energy (12.66%) and degradation/utilization/assimilation of inorganic nutrient metabolism (12.2%) (Fig. 2).

**Table 1:** Summary statics table.

Reads	Count
Total analyzed reads	190,953
Classified reads	190,918
Unclassified reads	35

## 2 Experimental Design, Materials and Methods

### 2.1 Sample collection

A 5–30 cm deep soil sample (about 300 g) was collected from Yok Don national park in the Central Highlands, Vietnam, kept at 4°C, and transported to the laboratory within 2 h. The sample was stored at −80°C until analyzed.

### 2.2 DNA extraction and the 16S rRNA gene amplicon sequencing

DNA was extracted from 0.3 g of the soil sample using the DNeasy PowerSoil kit (Qiagen, Germany). The V1–V9 region of the 16S rRNA gene was amplified from the extracted DNA. Libraries of the 16S rRNA gene amplicon were prepared using the Swift amplicon 16S plus internal transcribed spacer panel kit (Swift Biosciences, USA) according to the manufacturer's instructions. The 16S rRNA gene amplicon sequencing was performed using the Illumina MiSeq platform (2 × 150-bp paired ends). Primers used for amplification are shown in Table 2.

**Table 2:** Primers used for amplification in this study.

Primer	Sequence (5′‒3′)
F1	GAGTTTGATCMTGGCTCAG
F2	CCTACGGGAGGCAGCAG
F3	GCCAGCAGCCGCGGTAA
F4	ATGGCTGTCGTCAGCT
F5	GYAACGAGCGCAACCC
R1	CTACCAGGGTATCTAATCC
R2	CCGTCAATTCMTTTGAGTTT
R3	GACGGGCGGTGTGTACAA
R4	TACCTTGTTACGACTT

**Note:** F, forward primer; R, reverse primer.

### 2.3 Taxonomic and functional analyses

Taxonomic analysis was performed as described previously [6]. Briefly, the raw basecall (bcl) files were demultiplexed using bcl2fastq, allowing one mismatch in the dual-barcode sequence. Trimmomatic (v.0.39) [7] and Cutadapt (v.2.10) [8] were used to remove adapters, primers, and low-quality sequences (average score: < 20; read length: < 100 bp). The q2-dada2 plugin and denoise-single method within the QIIME2 pipeline (v.2020.8) [9] were used to cluster and dereplicate the reads into amplicon sequence variants. The QIIME2 aligned with the SILVA SSURef reference database (v.138) [10] was used for the taxonomic analysis of the amplicon sequence variants according to the classify-consensus-blast method [11]. Finally, the PICRUSt2 (v.2.3.0-b) [12] and MetaCyc databases [13] were used to predict the functional profiles of the soil sample based on the 16S rRNA gene amplicon sequencing. The analyzed functional profiles included degradation/utilization/assimilation, biosynthesis, super pathways, precursor metabolite and energy generation, detoxification, glycan pathways, metabolic clusters, macromolecule modification, and activation/Inactivation/Interconversion.

## Ethics Statements

None

## CRediT authorship contribution statement

**Dinh Minh Tran:** Conceptualization, Methodology, Software, Data curation, Writing – original draft, Investigation, Formal analysis, Validation, Visualization, Writing – review & editing. **To Uyen Huynh:** Investigation, Formal analysis. **Thi Huyen Nguyen:** Investigation, Formal analysis. **Tu Oanh Do:** Investigation, Formal analysis. **Thi Phuong Hanh Tran:** Investigation, Formal analysis. **Quang-Vinh Nguyen:** Investigation, Formal analysis, Validation, Visualization. **Anh Dzung Nguyen:** Investigation, Formal analysis, Validation, Visualization, Writing – review & editing.

## Declaration of Competing Interest

The authors declare that they have no known competing financial interests or personal relationships that could have appeared to influence the work reported in this paper.
